# Supplementary material for: Design, Synthesis, and Biological Evaluation of 2-Mercaptobenzoxazole Derivatives as Potential Multi-Kinase Inhibitors
Source: Pharmaceuticals (Basel). 2023 Jan 9;16(1):97. doi: 10.3390/ph16010097 (PMC9863562; doi:10.3390/ph16010097)
Supplement: Supplementary file 1 [file pharmaceuticals-16-00097-s001.zip › pharmaceuticals-2116216-supplementary.pdf]

Design, Synthesis and Biological Evaluation of 2-Mercaptobenzoxazole Derivatives as Potential Multi-kinase Inhibitors

Mohammed M. Alanazi[1]*, Saleh Aldawas[1], Nawaf A. Alsaif[1]

[1] Department of Pharmaceutical Chemistry, College of Pharmacy, King Saud University, Riyadh 11541, Saudi Arabia
* Correspondence: mmalanazi@ksu.ed

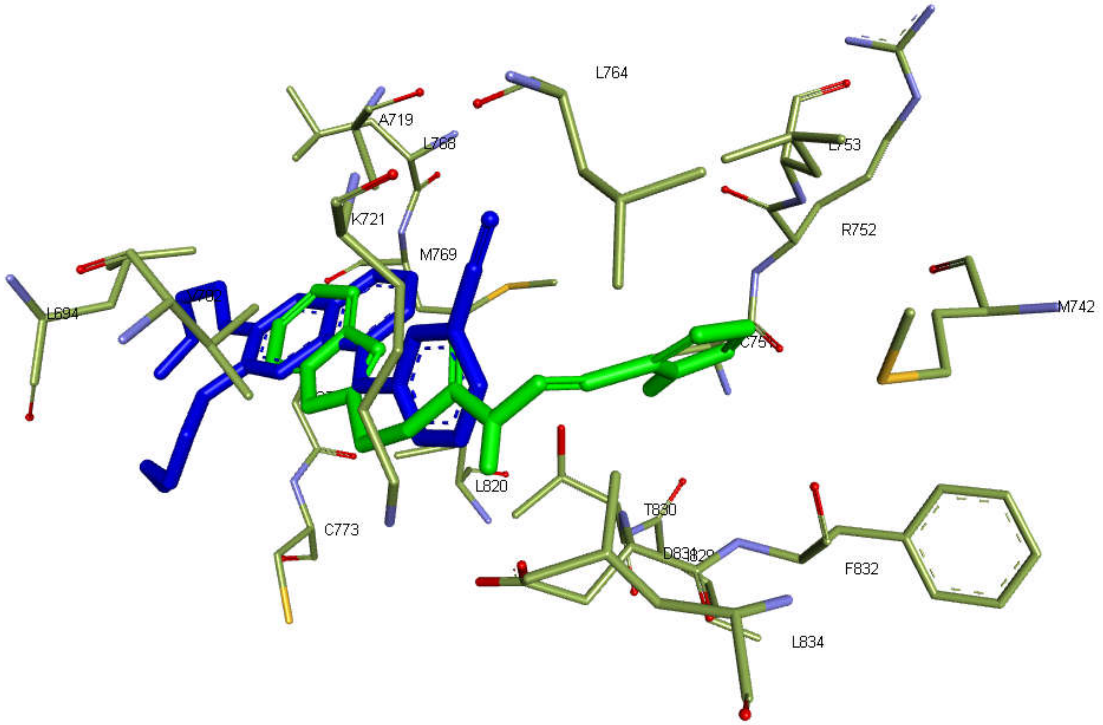

Figure S1: superimposition of erlotinib (blue) and compound 6b (green) in the active site EGFR.

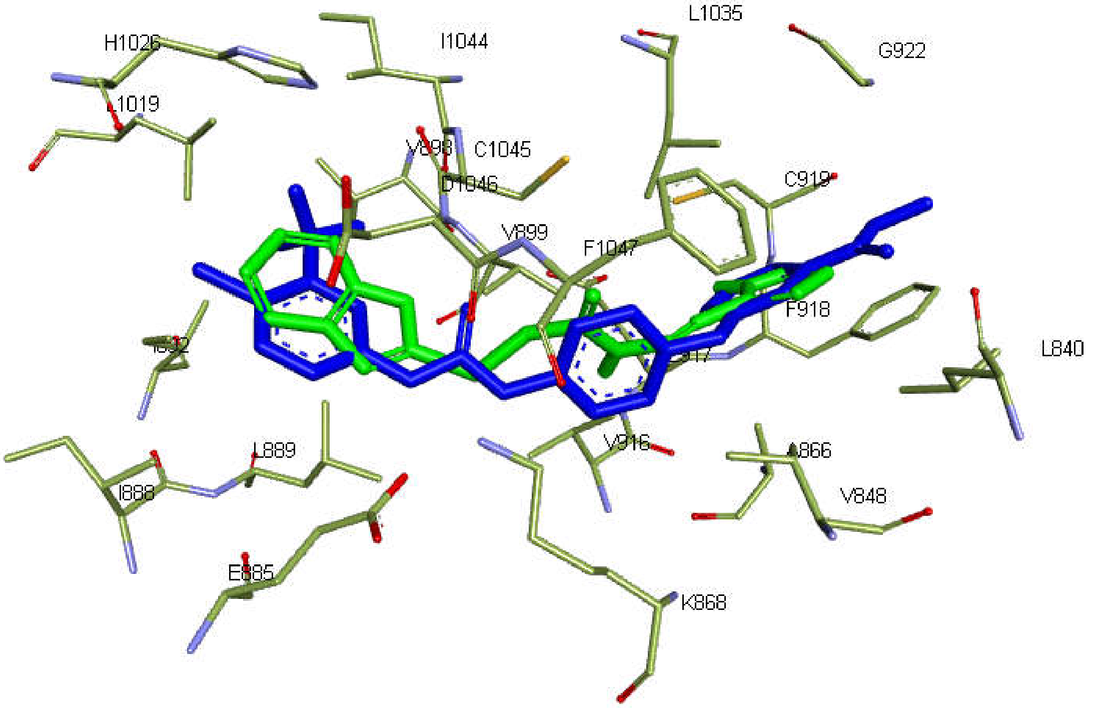

Figure S2: superimposition of sorafenib (blue) and compound 6b (green) in the active site VEGFR2.

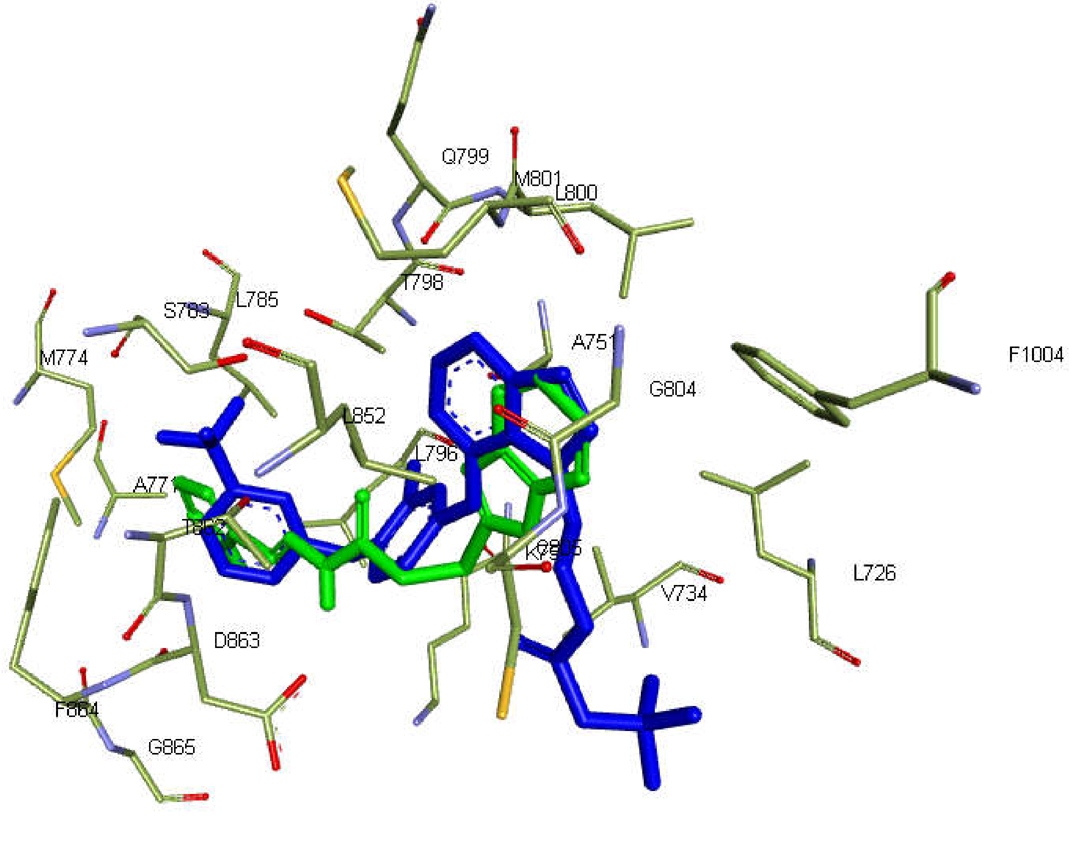

Figure S3: superimposition of lapatinib (blue) and compound 6b (green) in the active site HER2.

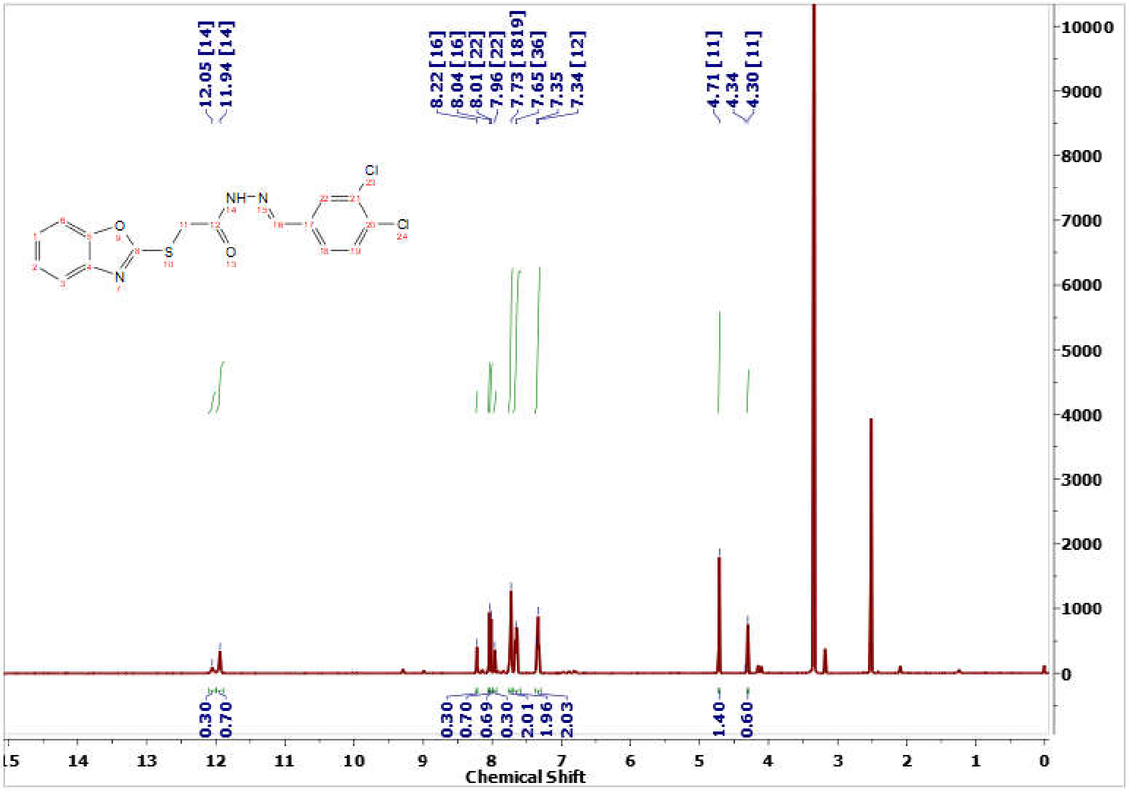

Figure S4: ^{1}HNMR of compound 4a

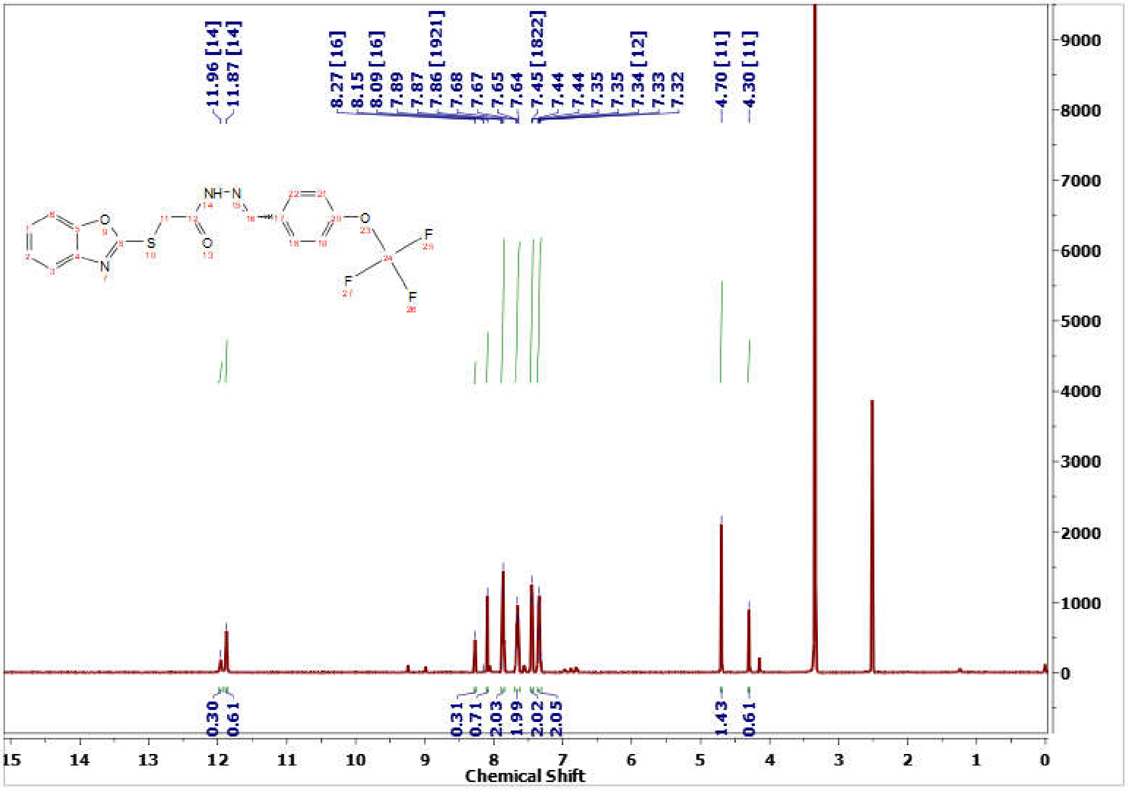

Figure S5: ^{1}HNMR of compound 4b

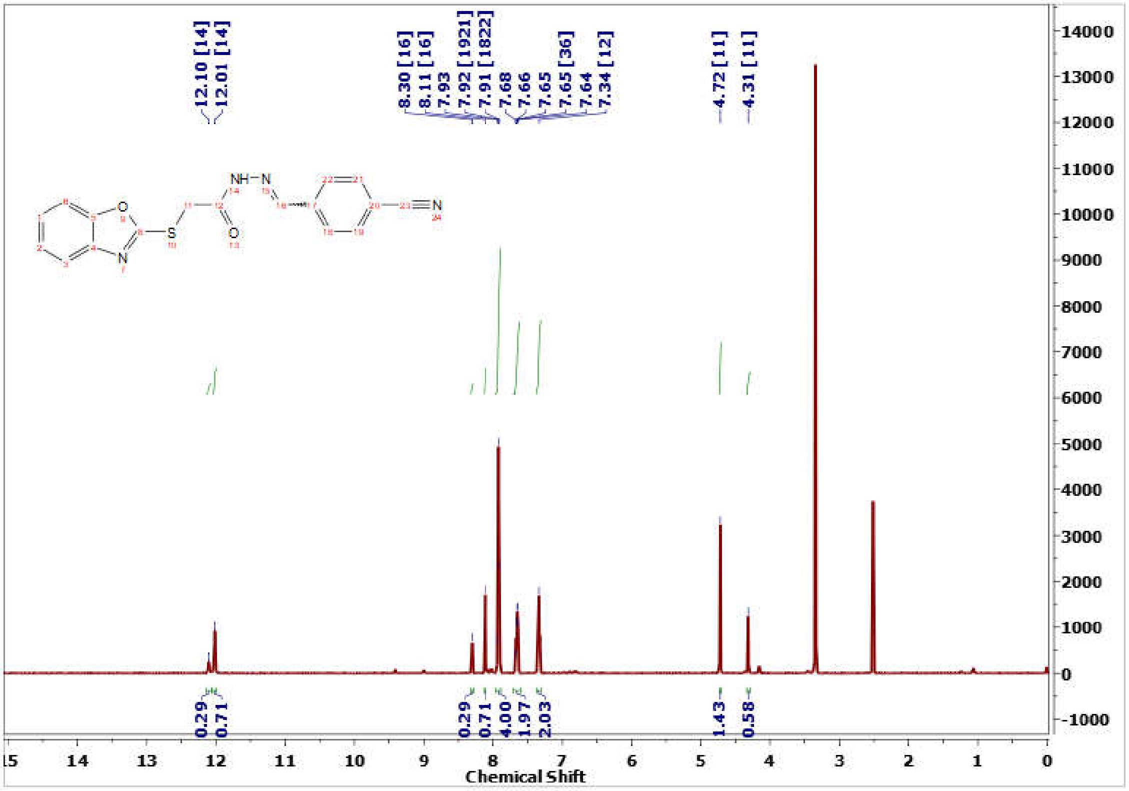

Figure S 6: 1HNMR of compound 4c

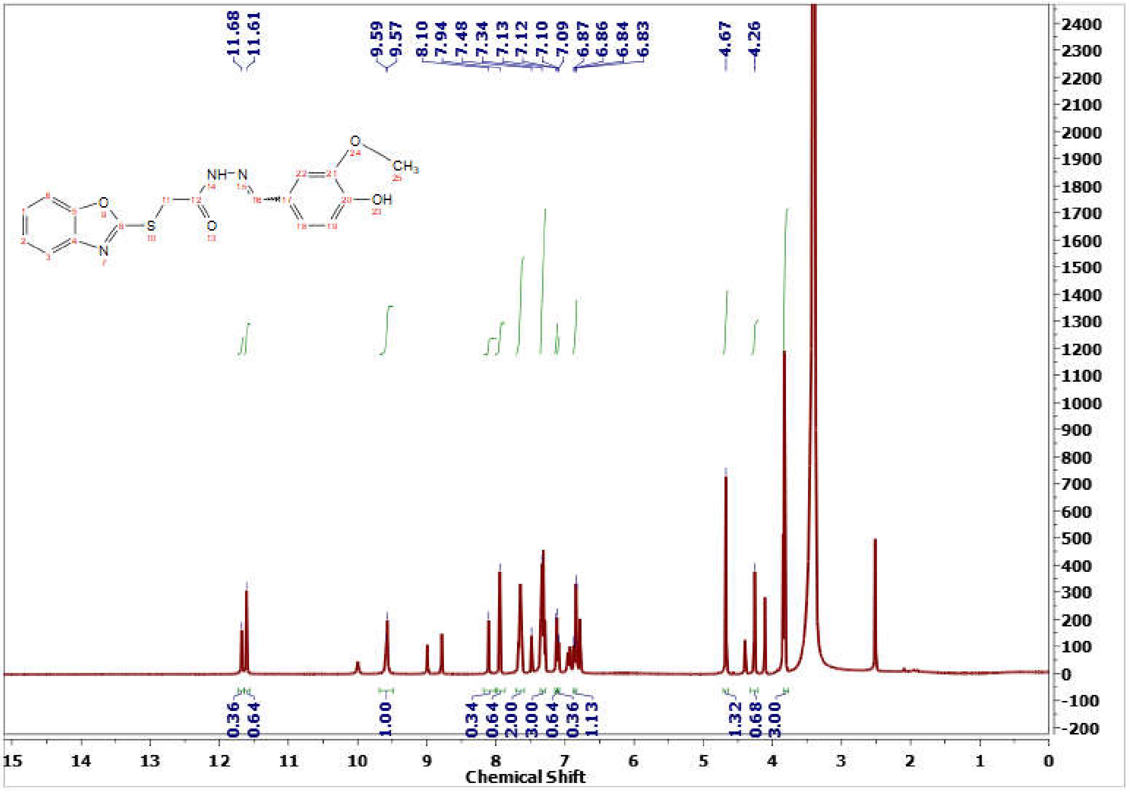

Figure S7: 1HNMR of compound 4d

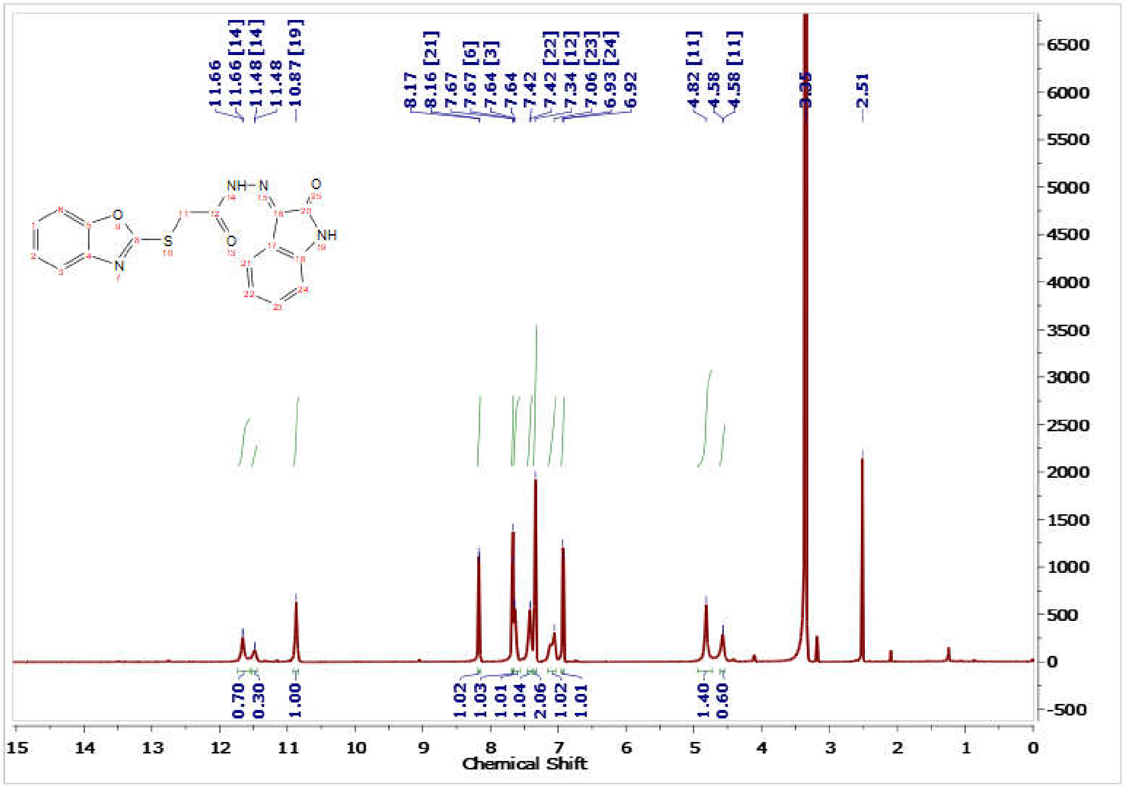

Figure S8: ^{1}HNMR of compound 5a

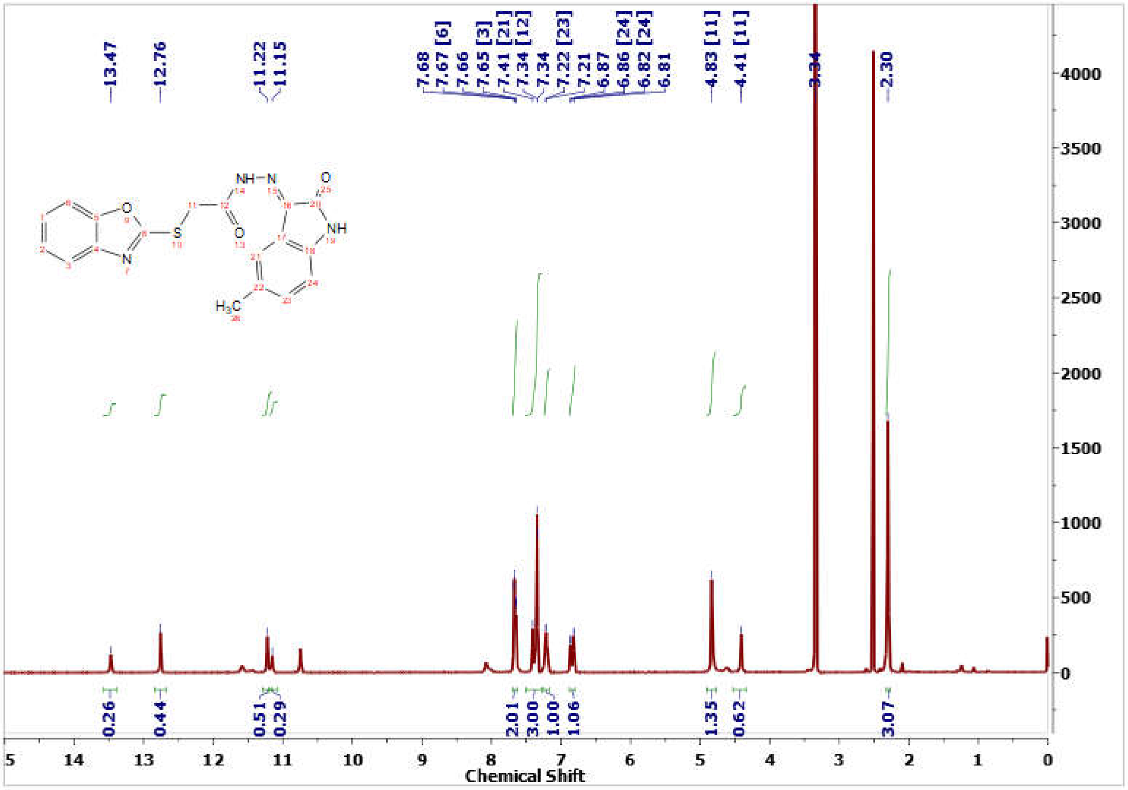

Figure S9: ^{1}HNMR of compound 5b

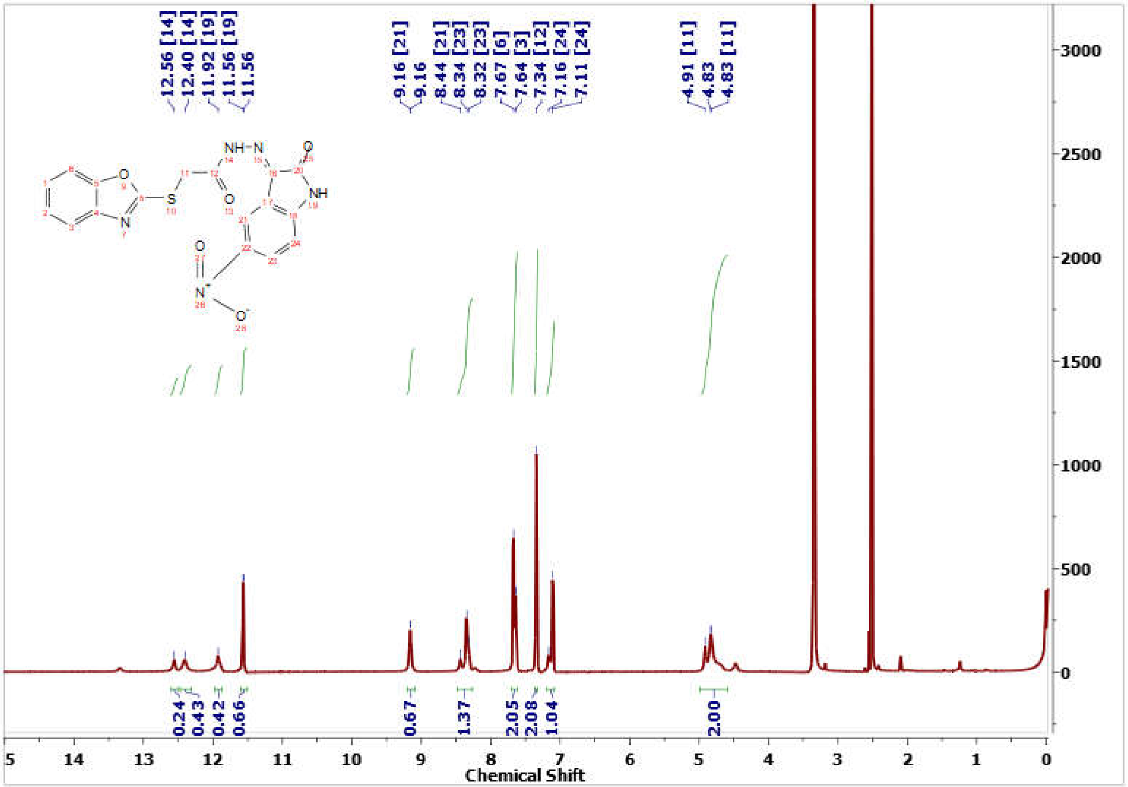

Figure S10: ¹HNMR of compound 5c

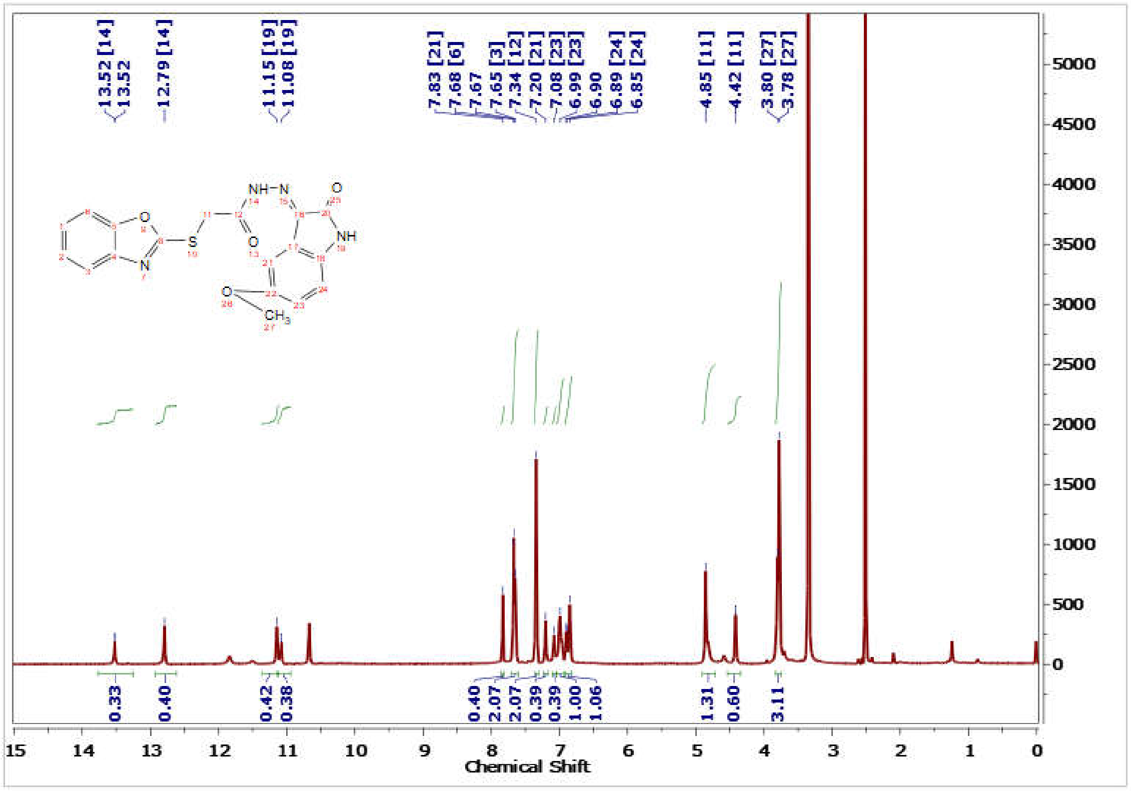

Figure S11: ¹HNMR of compound 5d

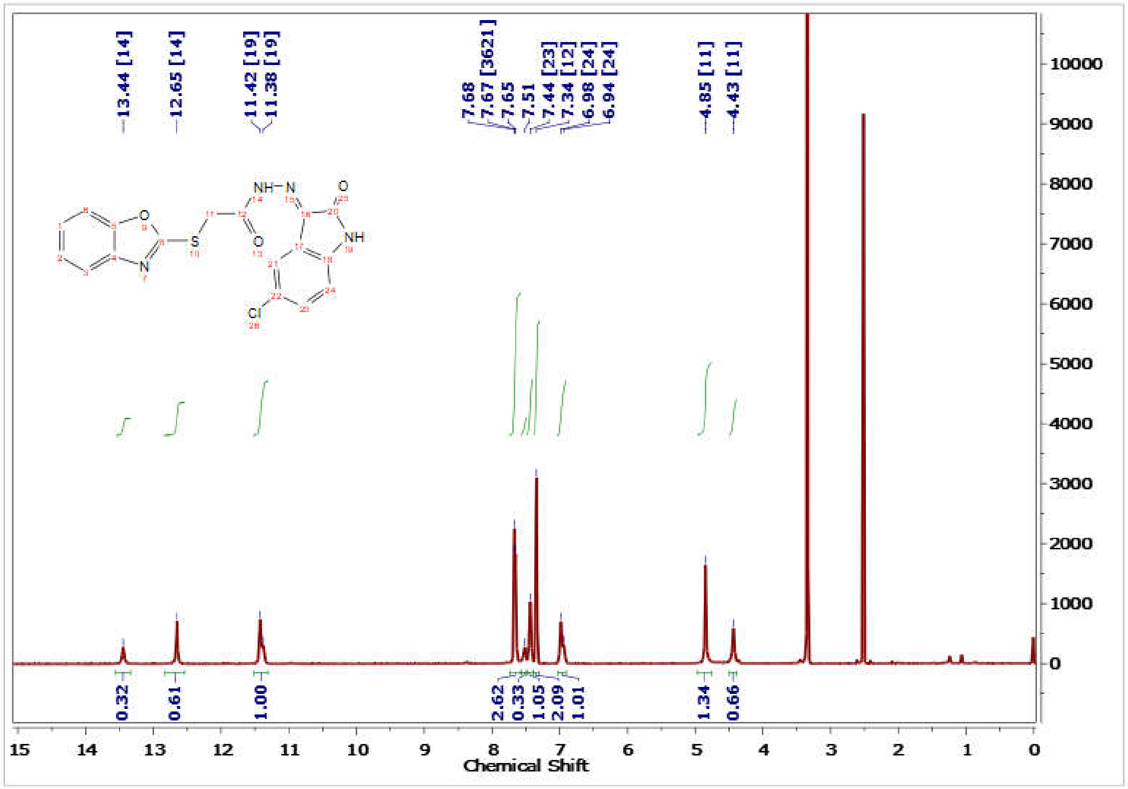

Figure S12: ^{1}HNMR of compound 5e

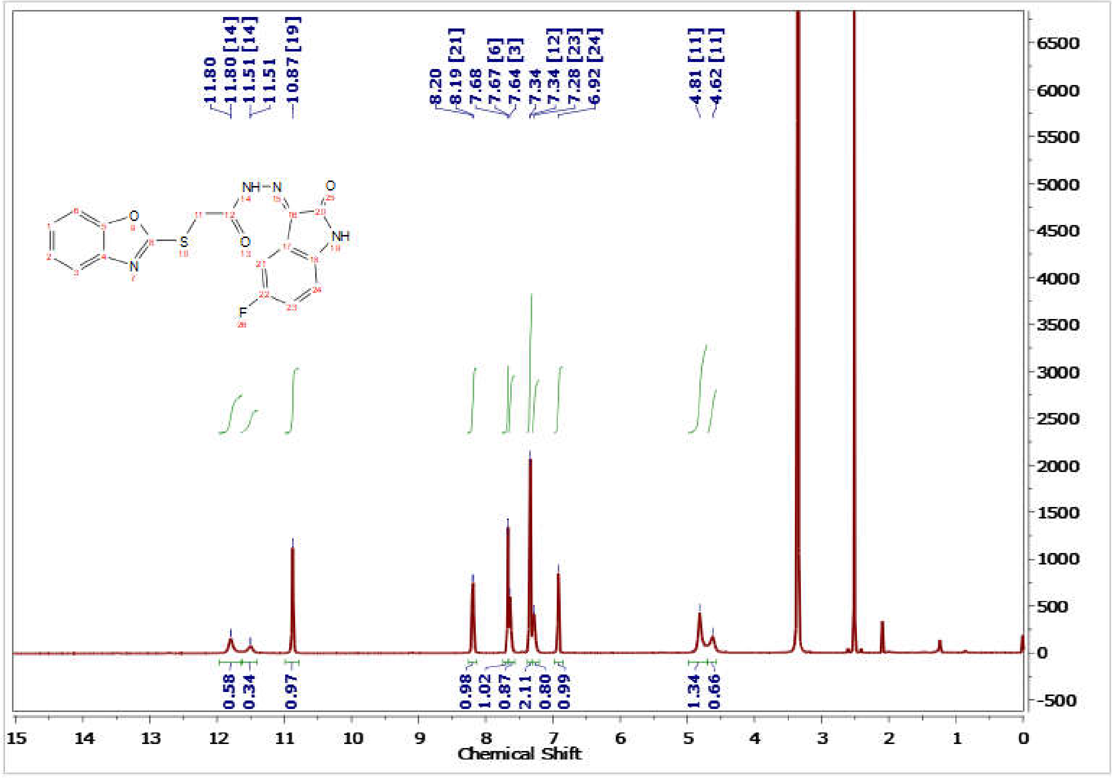

Figure S13: ^{1}HNMR of compound 5f

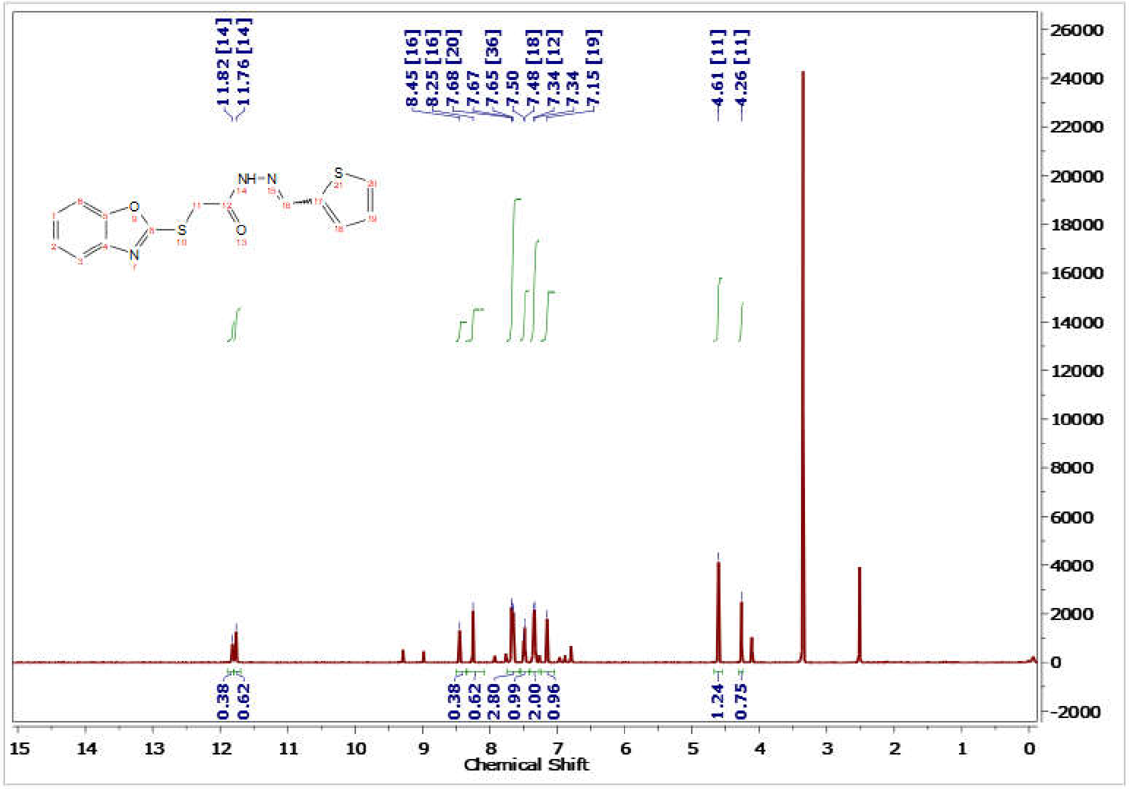

Figure S14: ^{1}HNMR of compound 6a

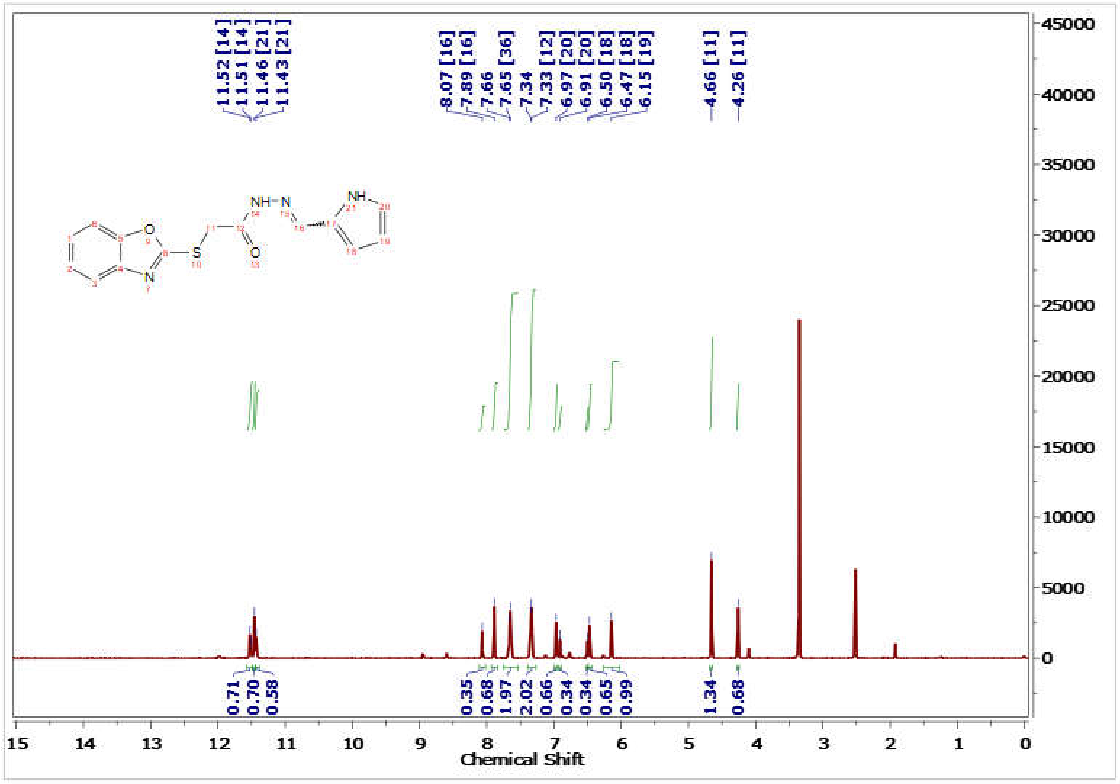

Figure S15: ^{1}HNMR of compound 6

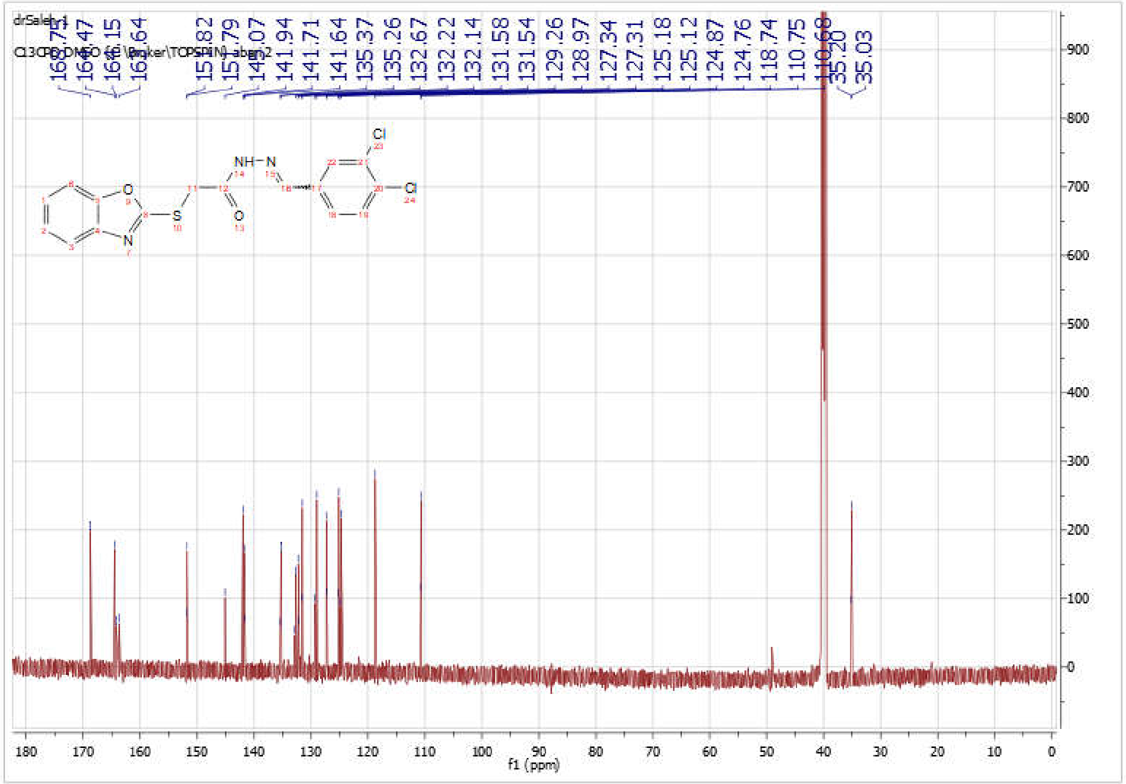

Figure S16: ^{13}CNMR of compound 4a

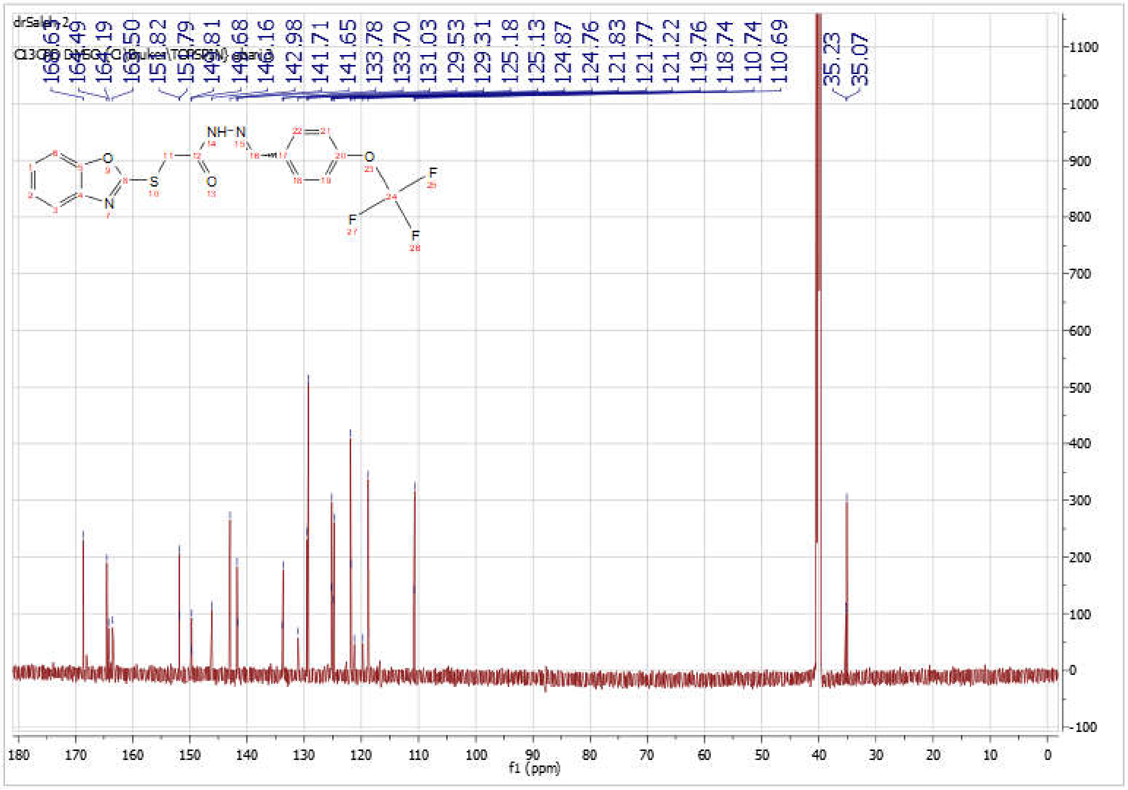

Figure S17: ^{13}CNMR of compound 4b

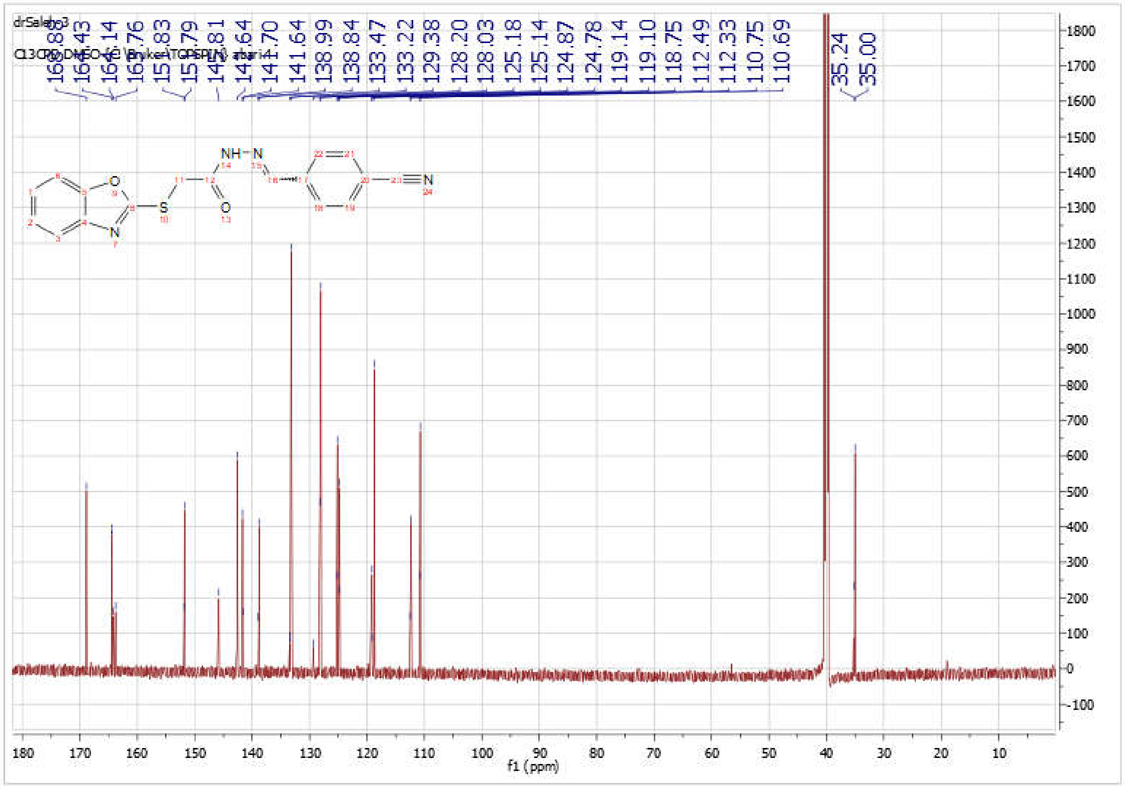

Figure S18: ^{13}CNMR of compound 4c

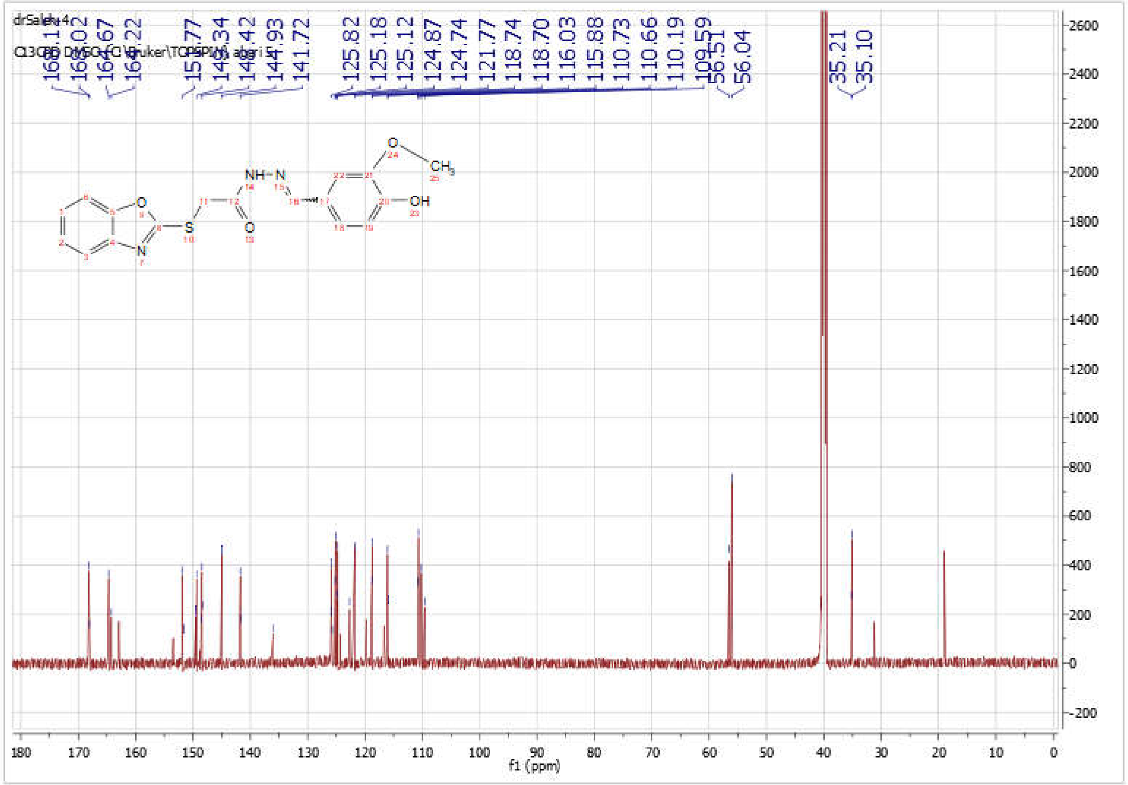

Figure S19: ^{13}CNMR of compound 4d

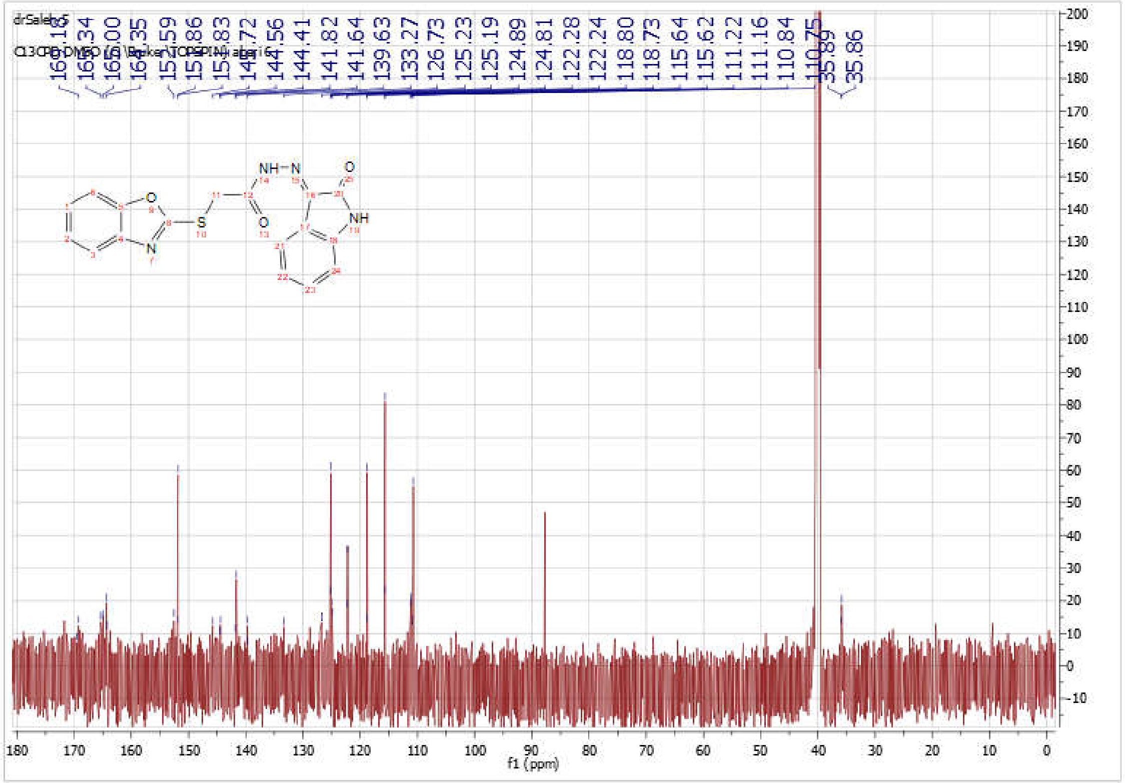

Figure S20: ^{13}CNMR of compound 5a

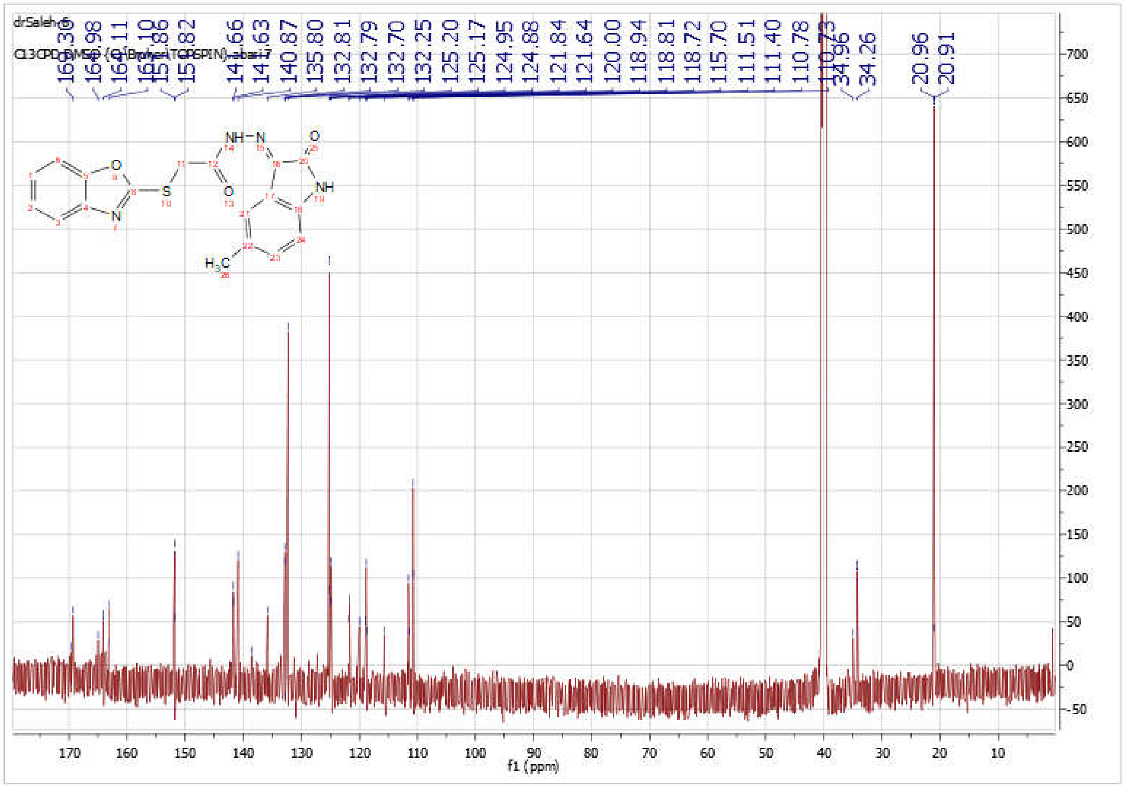

Figure S21: ^{13}CNMR of compound 5b

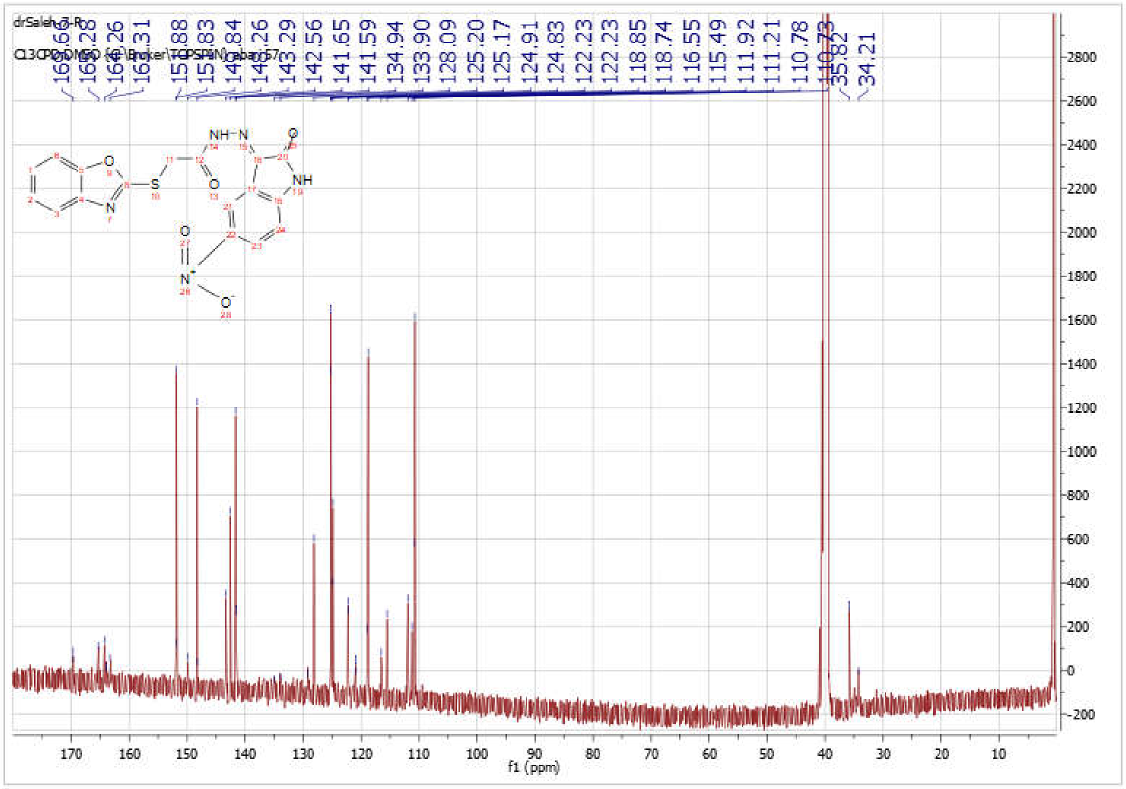

Figure S22: ^{13}CNMR of compound 5c

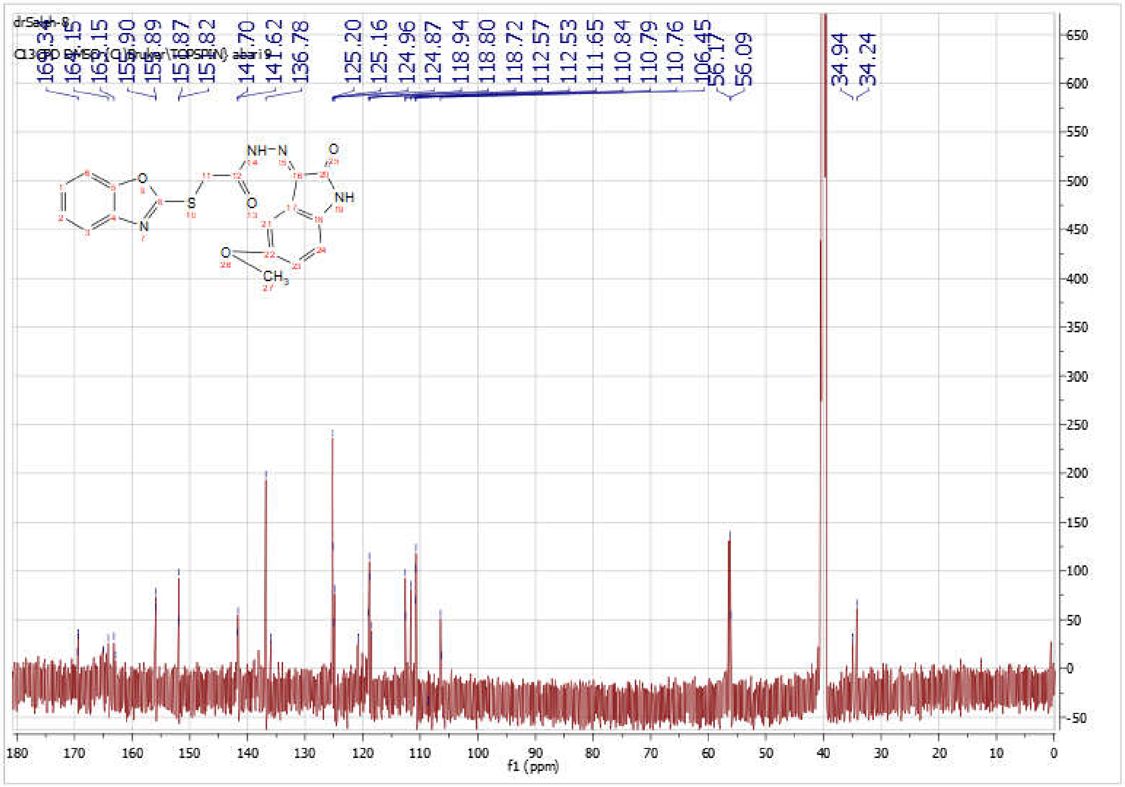

Figure S23: 13CNMR of compound 5d

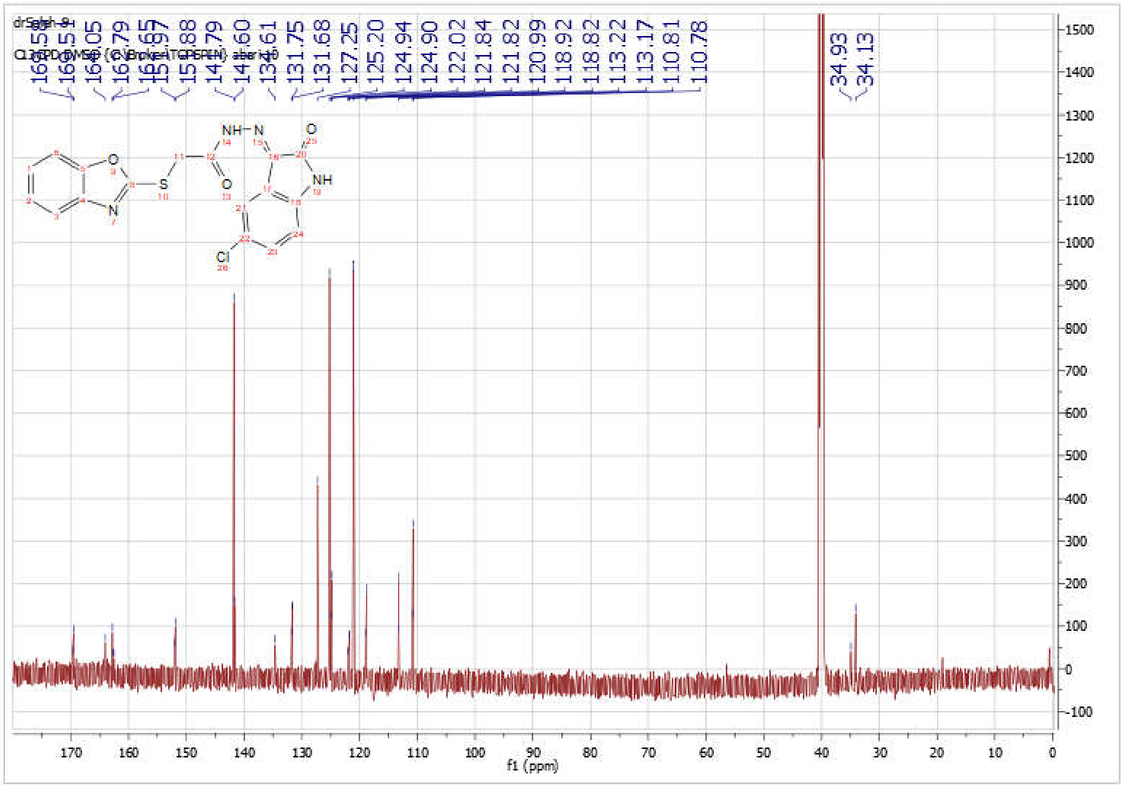

Figure S24: ^{13}CNMR of compound 5e

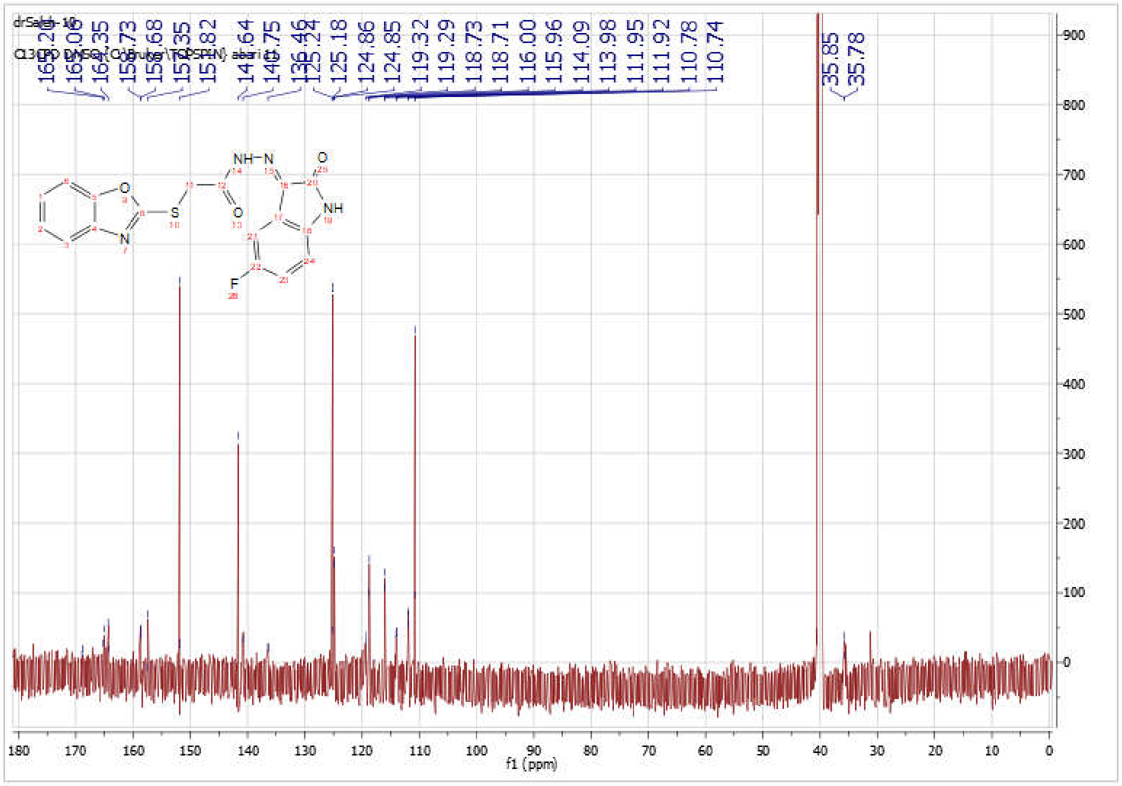

Figure S25: ^{13}CNMR of compound 5f

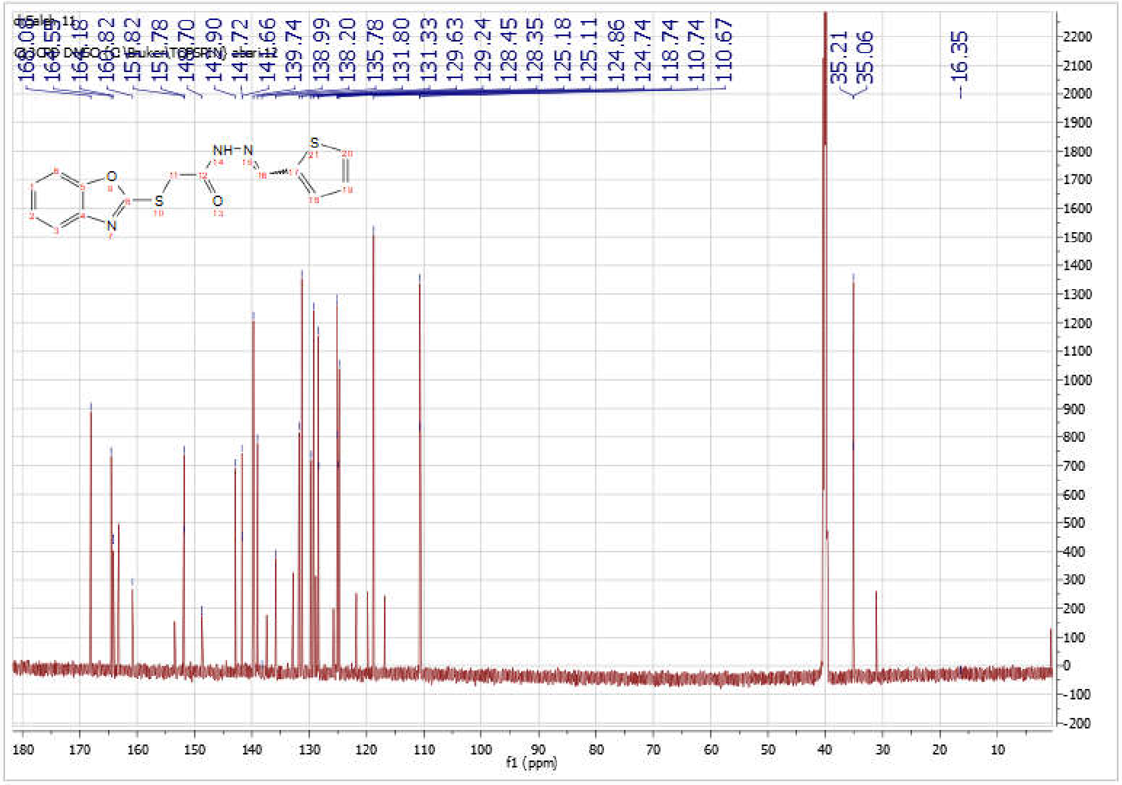

Figure S26: ^{13}CNMR of compound 6a

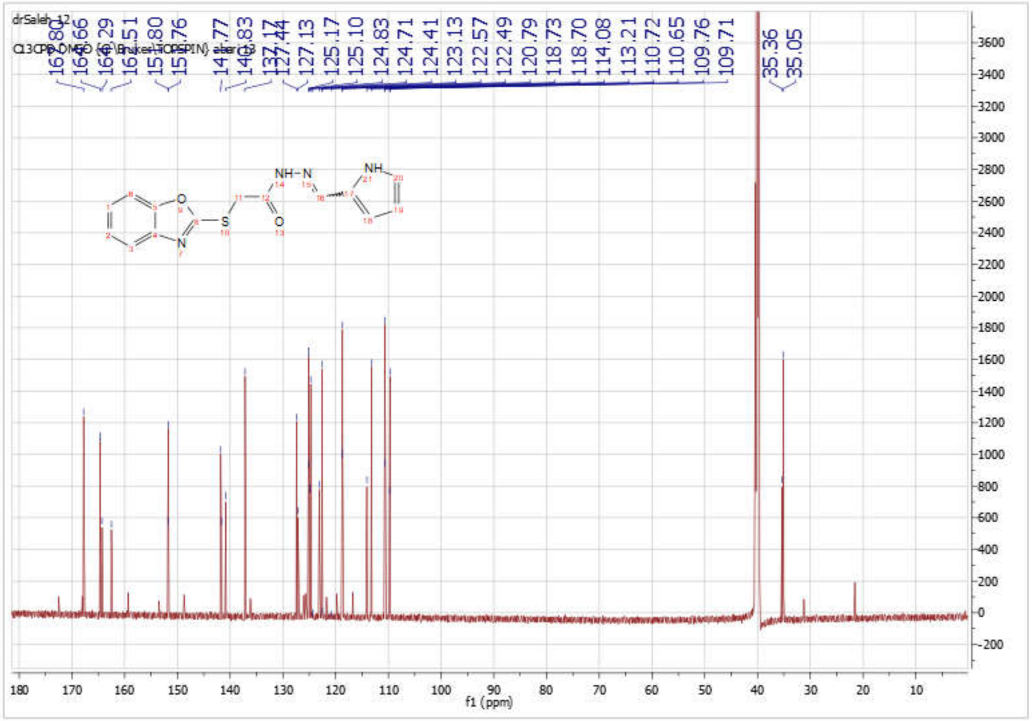

Figure S27: ^{13}CNMR of compound 6b

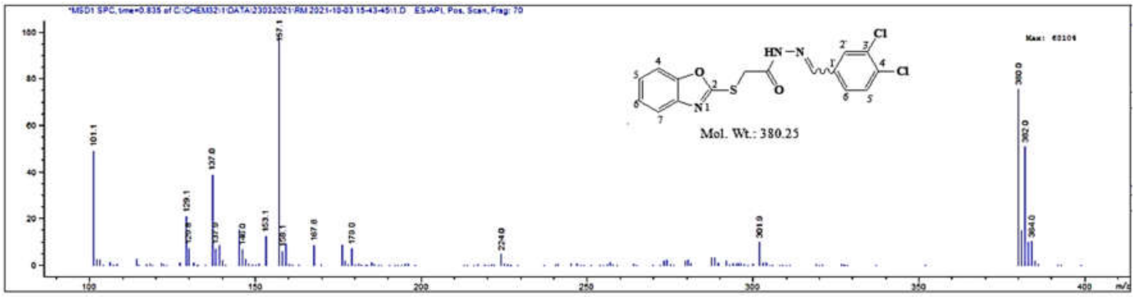

Figure S28: Mass of compound 4a

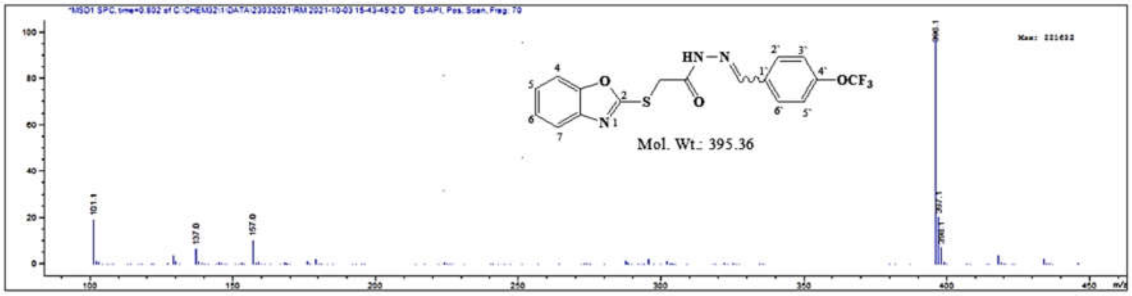

Figure S29: Mass of compound 4b

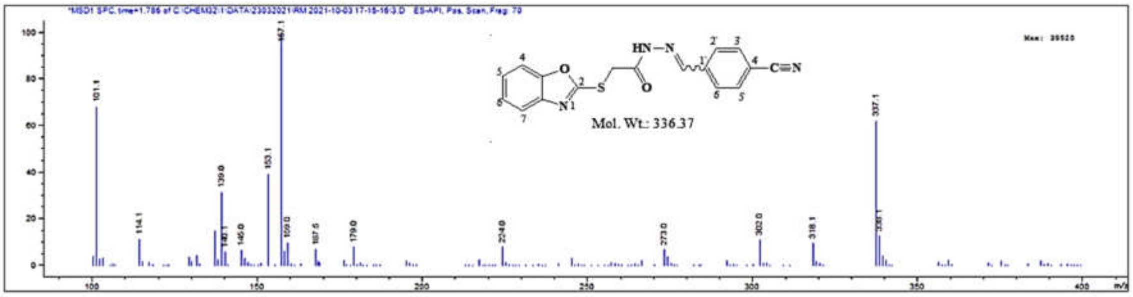

Figure S30: Mass of compound 4c

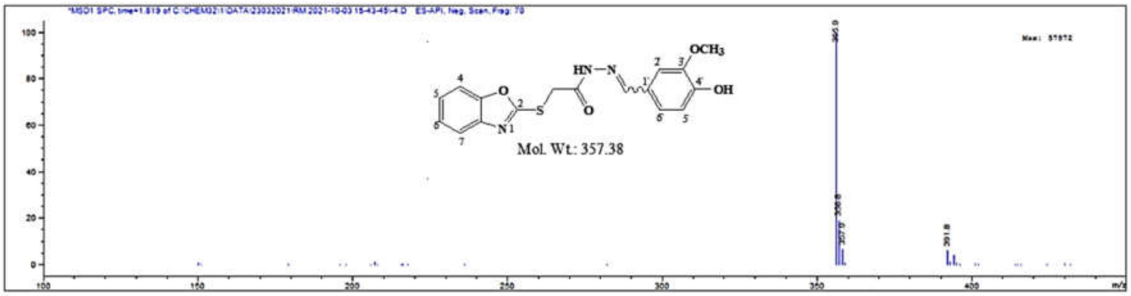

Figure S31: Mass of compound 4d

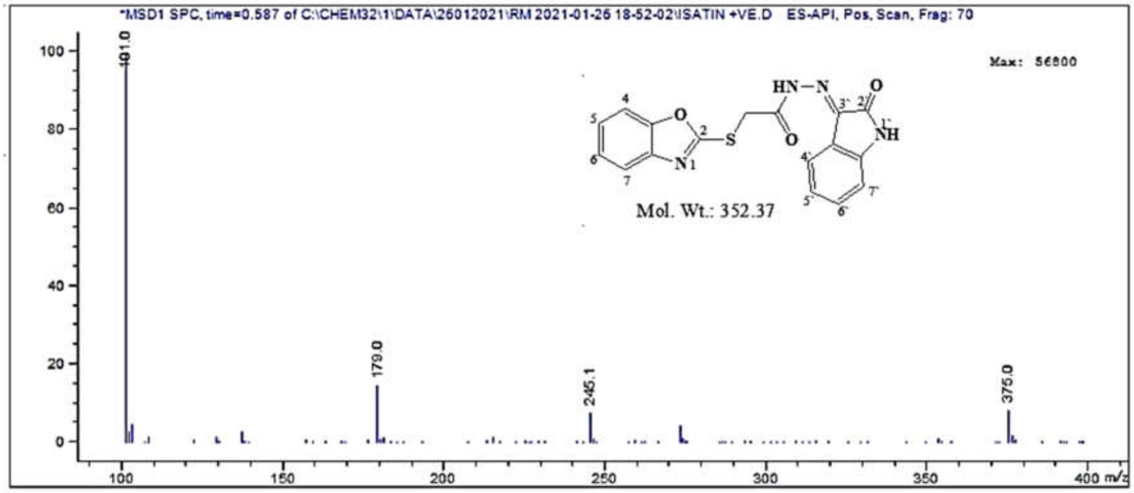

Figure S32: Mass of compound 5a

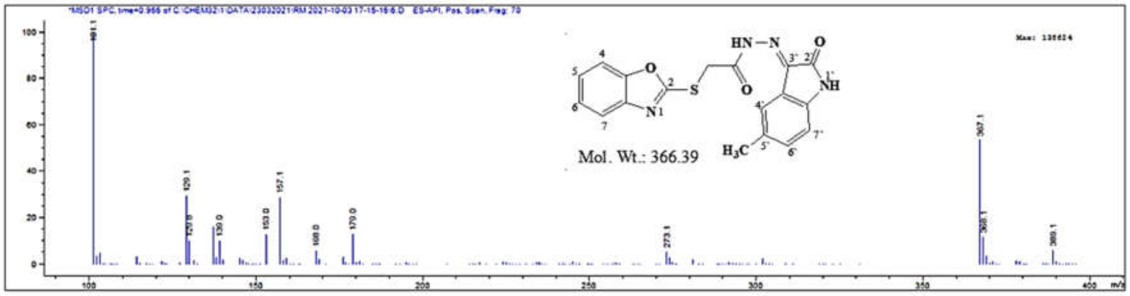

Figure S33: Mass of compound 5b

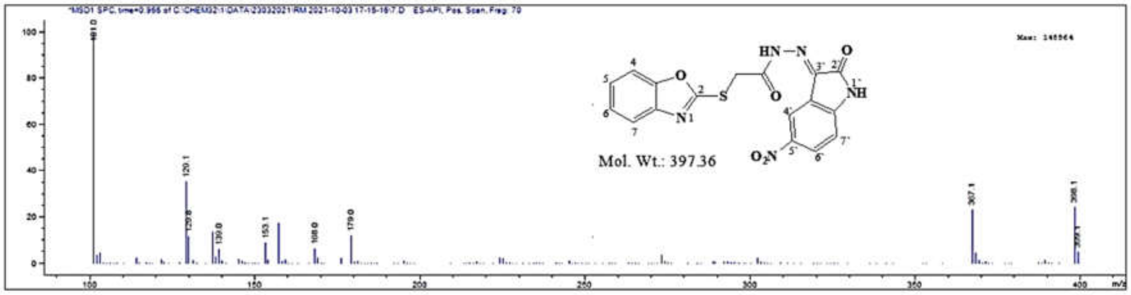

Figure S34: Mass of compound 5c

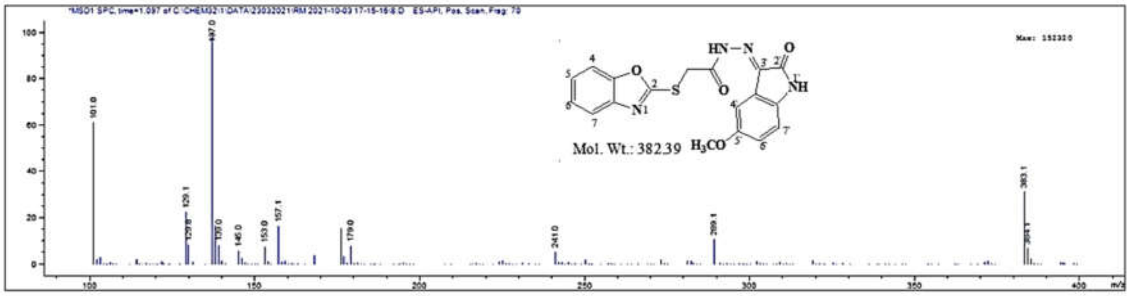

Figure S35: Mass of compound 5d

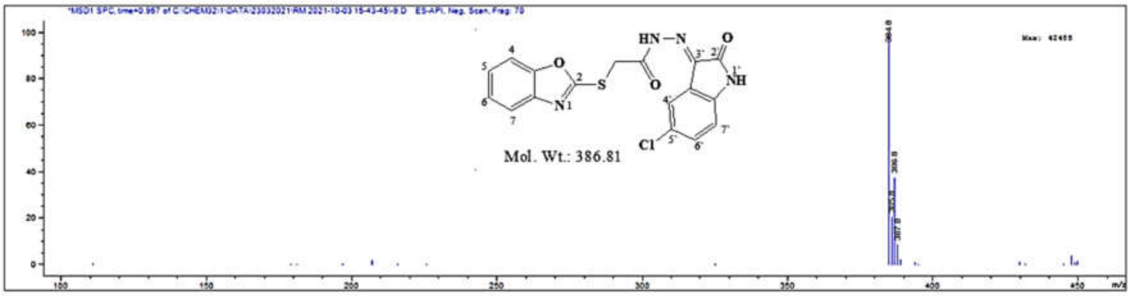

Figure S36: Mass of compound 5e

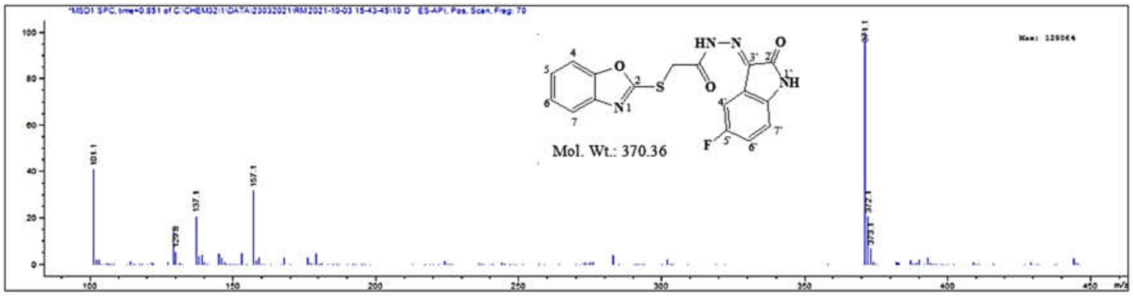

Figure S37: Mass of compound 5f

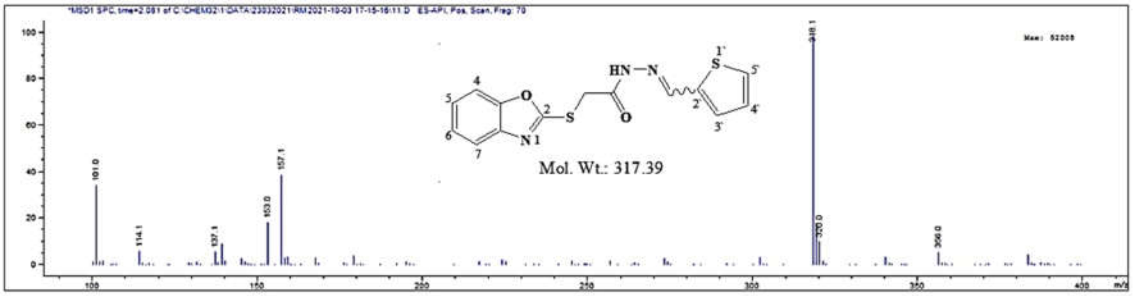

Figure S38: Mass of compound 6a

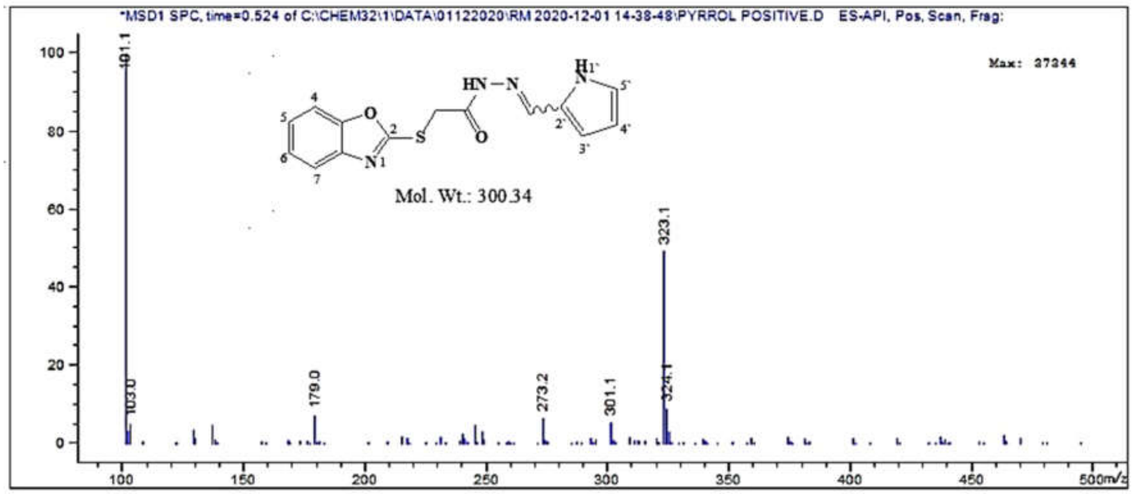

Figure S39: Mass of compound 6b
